# Decision-Making of Swiss Farmers and the Role of the Veterinarian in Reducing Antimicrobial Use on Dairy Farms

**DOI:** 10.3389/fvets.2020.00565

**Published:** 2020-08-26

**Authors:** Manuela Gerber, Salome Dürr, Michèle Bodmer

**Affiliations:** ^1^Vetsuisse-Faculty, Clinic for Ruminants, University of Berne, Berne, Switzerland; ^2^Vetsuisse-Faculty, Veterinary Public Health Institute, University of Berne, Berne, Switzerland

**Keywords:** antimicrobial reduction, farm veterinarians, motivators, management changes, influence

## Abstract

The reduction of antimicrobials on dairy farms is a topical issue and confronts both veterinarians and farmers with major challenges. The aim of this study was to investigate dairy farmers' motivation to reduce antimicrobial use on their farms. Factors influencing dairy farmers' decision-making regarding dairy cow health were identified and the role of the veterinarian in these processes was characterized. A customized structured questionnaire was sent to all participants (*n* = 59) of an ongoing antimicrobial reduction project among dairy farmers in the Canton of Fribourg, Switzerland, by mail. Fifty-eight completed questionnaires were returned and evaluated (response rate 98.3%). The majority of respondents were men (56/58, 96.6%) and farm managers (55/57, 96.5%) managing their farms as their main occupation (56/57, 98.2%). Using a 5-point-Likert-scale (1 = not a reason, 5 = very important reason), respondents ranked “My veterinarian is putting pressure on us to use less antimicrobials” (median=2.5, interquartile range = 1–3) and “Other farmers also reduce antimicrobial use” (2.0, 1–3) as the least important factors affecting their motivation to reduce the use of antimicrobials in dairy cows (*P* < 0.001). Respondents ranked their veterinarian's opinion (4.0, 4–5) and their own feelings and knowledge (4.0, 3–4) as the two factors having significantly more importance on their decisions regarding dairy cow management (*P* < 0.001). The farmers indicated they were satisfied with the quality of the consultancy given by their veterinarians (4.0, 4–5) and with the quality of communication with veterinarians (4.0, 3–4). They indicated that they understood recommendations made by the veterinarian (4.0, 3.75–4) and also felt understood by the veterinarian (4.0, 3–4). However, only 25.9% (14/54) indicated they were willing to pay for good quality, farm-adapted consulting by their veterinarian. Based on these findings, veterinarians play an important role in influencing Swiss dairy farmers in decision-making concerning animal health and treatment. However, veterinarians were not viewed by farmers as important motivators for reducing antimicrobial use. Swiss veterinarians are encouraged to be aware of their influence on farmers' decisions and to use that influence to more clearly promote antimicrobial reduction on dairy farms.

## Introduction

The reduction of antimicrobial use in veterinary medicine is important since antimicrobial resistance is a concern for animal and public health (1). Increasing milk production in dairy cows is associated with production diseases (e.g., mastitis or metritis) that increase the use of antimicrobials (2, 3). Antimicrobial use leads to an increase of multidrug-resistant bacteria, while at the same time the development of new (non-resistant) antimicrobials is lacking (4). A restrictive, targeted, and evidence-based use of antimicrobials is therefore crucial. For this reason, antimicrobial use has been strictly regulated by law in many countries. Restriction by law is also recommended by the Food and Agriculture Organization of the United Nations (FAO) to enhance the sustainability of policy and technical objectives, and to clarify the roles and responsibilities of governments and other stakeholders (5). Veterinarians are obliged to reduce antimicrobial use, but their prescribing behavior may be influenced by many different factors (6, 7).

The influence of farmers on veterinarians is also an important consideration with regard to antimicrobial use. Various socio-psychological models, for example the theory of planned behavior or the RESET Mindset model, explain that attitude, knowledge, and motivation strongly influence human behavior (8–10). According to the theory of planned behavior, a certain behavior is determined by the intention to perform this behavior. For example, the request of farmers for antimicrobial prescriptions is controlled by their intent to administer the drugs to their animals. This intention is in turn influenced by various factors, including behavioral beliefs and normative beliefs (9). Behavioral beliefs are those in which a positive attitude toward a behavior is more likely to lead to its implementation. The greater the understanding of the benefits of a certain behavior, or the greater the motivation to carry out this behavior, the more positive the attitude toward this behavior. Normative beliefs reflect the presumed expectation that persons close to the acting person, such as neighbors or family members, are executing a certain behavior.

Fischer et al. (11) stated that to understand the decision-making process, “we need to understand not only farmers” behavior *per se* but also the underlying reasons and the context this behavior occurs in'. Therefore, the attitude toward a certain behavior as well as social pressure and the knowledge of a person play important roles in the actual execution of a behavior (8, 12, 13). Jansen et al. (14) showed that if the attitude, knowledge, and behavior of dairy farmers toward a specific topic was changed (e.g., improved hygiene during the milking process) udder health was improved in the long term.

To achieve a behavioral change that leads to new actions for improving animal health, many factors must be taken into account, including the technically correct implementation of an action (15–17). Often, the unsuccessful implementation of management changes in practice is caused not by demanding technical aspects of the new management, but by the attitude, motivation, and knowledge of farmers, or social pressure from outside (18). For veterinarians to motivate farmers and successfully encourage or advise them to change a certain behavior, individual factors must be addressed. Lack of knowledge and insufficient support can lead to unsuccessful implementation in practice (19, 20). Notably, economic advantages such as increased profitability and enhanced job satisfaction can promote positive attitudes (12).

As many practicing veterinarians have already experienced, evidence-based strategies for reducing antimicrobial use and the associated management changes are not as easy to implement on commercial farms as under well-defined study conditions. It has been suggested that the motivation of Swiss dairy farmers plays an important role in their willingness to implement recommendations made by their veterinarian (21). This motivation in turn was found to be influenced by the relationship between the veterinarian and the farmer and by the support the veterinarian provided to the farmer (22). There is some evidence that farmers with no intent to adopt control measures identified their veterinarian as the “preferred motivator,” whereas for farmers with the intent to adopt control measures, consumer demand, and financial rewards or penalties were significant motivators (19). However, it is not easy for veterinarians to establish a relationship with the farmer that provides optimal support (23).

In the pig industry, differences in the use of antimicrobial drugs between countries are caused mainly by differences in legislation (24–27). However, there is a lack of such comparative studies in the dairy sector. The motivations and attitudes of Swiss dairy farmers toward the reduction of antimicrobial use and the implementation of management changes aimed at disease prevention are currently unknown. In contrast to other EU countries, Switzerland is a highly subsidized agricultural setting with very high milk quality requirements. Some researchers hypothesized that the drivers for prescribing antimicrobials in veterinary medicine were similar to those in human medicine, including incorrect knowledge and the expectation of the patient (client) to be prescribed medication (28–31). For Swiss livestock veterinarians, various extrinsic and intrinsic factors are important in the decision to use antimicrobials, and veterinarians play an important role in their use and thus reduction (7). However, it is also known that communication between farmers and veterinarians is often challenging (18, 22). For veterinarians it is important to know how to motivate their farmers in order to achieve successful changes in dairy cow management and to support farmers in improving animal health. In this study we aimed to investigate dairy farmers' motivation to reduce antimicrobial use on their farms and identify factors that influence them in deciding to reduce antimicrobial use by implementing preventive measure. Furthermore, the study aimed to describe the role of the veterinarian in the above mentioned areas from the farmers' point of view.

## Materials and Methods

### Study Population

A survey was conducted of dairy farmers in a subregion of Switzerland (canton of Fribourg) in December 2018. Fribourg is one of 26 cantons in Switzerland. It has an above-average proportion of dairy farms compared to other cantons (7.3%, 1,429 out of the 19,568 Swiss dairy farms) and contributes 9% of Swiss milk production (total >330,000 kg/year) (32, 33). With its flat, fertile landscapes, in which arable farming dominates, but also including mountainous regions, the canton is representative of the topography in Switzerland with regard to different working and management conditions for farmers. The study population included all farmers participating in the ReLait project, a voluntary program through which dairy farms can improve the health of dairy cows and replacement calves. The aim of the ReLait project is to reduce the use of antimicrobials in dairies by implementing evidence-based preventive measures consisting of management changes, without compromising the quality of animal products or animal health. The evidence-based management changes proposed by the ReLait project can be divided into three health sectors: udder health, uterine health, and calf health. Management changes within each sector are grouped into so-called “prevention strategies” to facilitate selection by farmers. Farmers who participate in the ReLait project had committed to implementing at least one of 17 suggested sets of management changes over a period of at least 2 years (January 2018–December 2019). The farmers were compensated for their participation in the project with CHF 500 (Swiss francs) annually. Furthermore, depending on the prevention strategies chosen, analyses (e.g., of milk samples) were also subsidized.

### Questionnaire Survey

A customized structured questionnaire was sent to participating farmers by mail together with a pre-stamped return envelope. The questionnaire was available in German and French, respecting the different languages in the study region. The questionnaire was pre-tested by three farmers not involved in the project, to check comprehensibility and, if necessary, to supplement missing response options. After 1 month, a reminder was sent to all farmers by email. Those who had not returned the questionnaire by the deadline were called by the project team and asked to return the completed questionnaire. There was no incentive for completing the questionnaire apart from the rewards for participation in the ReLait project described above.

The questionnaire consisted of five sections: Part I. Demographic data; Part II. Decision-making process of the farmer; Part III. Motivation for reduction of antimicrobial use; Part IV. Motivation for selection of prevention strategies; and Part V. Relationship to their veterinarian. For the current study 13 questions were evaluated (Table 1); other data collected in the context of the ReLait project were not relevant for the present study. Together with the questionnaire, a guideline was sent on how to complete the questionnaire to avoid misunderstandings or ambiguities.

**Table 1:** Survey questions and ranked responses used in this study.

**Part I. Demographic data**	
Question 1	Name
Question 2	Gender (male, female)
Question 3	Age
Question 4	Position on the farm (farm manager, employee, partner, or relative)
Question 5	How do you manage your farm? (main occupation, secondary occupation with <50% or ≥50% agriculture and farming)
Question 6	What is your main occupation? (dairy farming, beef/calf fattening, agriculture, other)
**Part II. Decision-making process**	
Question 7	To make important decisions (concerning management, animal health etc.) or to obtain important information… [rank each on a scale from 1 to 5 (1 = not a reason, 5 = very important reason)] •… the opinion of other farmers is important to me •… the opinion of my veterinarian is important to me •… I alone decide according to my feelings and knowledge •… this is discussed with the family •… I discuss with my employee •… I get help from “experts” in this field. •… I look for newspapers and corresponding articles to the topic
Question 8	What do you think of organized group meetings or farm visits together with other farmers? [rank each on a scale from 1 to 5 (1 = not a reason, 5 = very important reason)] • I can benefit from these meetings • I have tried tips from other farmers myself • I like to take part in such meetings • I do not like taking part because a lot of time is lost on my own farm • For me it is difficult to find out from the different opinions what is recommendable for me • I believe everything the other farmers say at these meetings
**Part III. Motivation for reduction of antimicrobial use**	
Question 9	What motivates you to reduce the use of antimicrobials? (Please give an assessment for each motivation reason) [rank each on a scale from 1 to 5 (1 = not a reason, 5 = very important reason)] • I would like to improve myself out of self-motivation • I would like to reduce costs for drugs • I have a certain responsibility to consumers • The news about the antimicrobial resistance problem motivates me • Sustainable, healthy production is important to me • A low use of antimicrobials corresponds to the rural tradition • A low use of antimicrobials corresponds to my personal idea of milk production • I want to improve the image of farmers • My vet is putting pressure on us to use less antimicrobials • Other farmers are also trying to reduce antimicrobials, which motivates me
**Part IV. Selection of prevention strategies**	
Question 10	Why did you decide on the specific prevention strategy “x”? [with “x” indicating the prevention strategy(ies) selected by the farmer] [rank each on a scale from 1 to 5 (1 = not a reason, 5 = very important reason)] • I have problems in this health sector on my own farm • I have the highest use of antimicrobials in this health sector on my farm • I am interested in trying something new • I want to approach this management change systematically and consistently, as I have never been consistent before • I am hoping for financial savings
**Part V. Relationship with the veterinarian**	
Question 11	With the quality of consultancy provided by my veterinarian I am in general: [rank on a scale from 1 to 5 (1 = very dissatisfied, 5 = very content)]
Question 12	For good quality, farm-adapted consultancy from the veterinarian…(select one of the statements below) •…I am willing to pay something in addition to treatments and drugs •…I am not willing to pay something; this belongs for me to the already paid service
Question 13	How do you rate the communication between your veterinarian and you? [rank each on a scale from 1 to 5 (1 = not a reason, 4 = very important reason)] • I feel understood • I understand his/her criticism and suggestions

All questions were asked in a closed form except for name and age. In some cases, a comment field was provided for entering free text. At the end of the questionnaire, respondents could write general comments or clarifications. Questions were followed by a list of response statements that respondents were asked to rank on a 5-point Likert scale in which 1 = not a reason (i.e., very dissatisfied) and 5 = very important reason (i.e., very content). Use of a 5-point ranking scale allows a statement to be judged as negative (1,2), positive (4,5), or neutral (3). Only question 13 was asked with a 4-point Likert scale so there was not a neutral answer option, requiring farmers to choose one side or the other.

### Farm Characteristics

Additional data to describe the study population were obtained from the above-mentioned ReLait project. To determine herd size, the average of all cows in the quarter October to December 2018 that had calved at least once by the end of December 2018 was calculated. Lactating and dry cows without heifers were included in the herd size calculation. To obtain data on the production label and location of each farm, farmers were interviewed in January–March 2018.

### Data Analysis

Statistical analysis was performed with R (Version 3.3.0, Boston, MA, USA; http://cran.r-project.org). Respondent age and herd size were reported as mean ± SD and range (minimum-maximum values). Other demographic data were reported as the number and percentage of respondents. Likert-like scale rankings were reported as median and interquartile range (IQR). Differences in the ranking of different factors were compared using a Kruskal Wallis test followed by pairwise comparisons using Wilcoxon-rank-sum test with the Benjamini and Hochberg correction as *post hoc* tests. To identify a difference in the factors motivating strategy selection (outcome variable) between the different health sectors (independent variable), a generalized linear mixed model was calculated with the farm ID as a random effect, as farmers had the possibility to choose several strategies per health sector.

## Results

Of 59 questionnaires sent out, 58 were returned and analyzed (response rate 98.3%). Not all questionnaires were answered completely, so the number of respondents for each question is reported.

### Demographic Data

Of the 58 farmers responding, 56 were men (96.6%) and one was a woman (1.7%); one respondent did not specify gender. Mean age was 47.7 ± 10.5 years (range 28–62 years). Most respondents were 50–59 years old (24/55, 43.6%), followed by 30–39 years (13/55, 23.6%), 40–49 years (8/55, 14.6%), 60–69 years (7/55, 12.7%%), and 20–29 years (3/55, 5.5%). The majority of the respondents were farm managers (55/57, 96.5%); 3.5% (2/57) were employees or partners or family members of the farm manager.

All but one respondents managed their farm as their main occupation (56/57, 98.2%). One (1.8%) farmer managed his farm as a secondary occupation (≥50% agriculture and farming). On 49 of 51 farms (96.1%) the main occupation was dairy farming. For two of the 51 farmers (3.9%), growing crops generated the main income.

The average herd size of farms was 46 ± 20 dairy cows (range 18–121 cows). Five of 58 farmers (8.6%) were producing under organic conditions (Bio-Suisse) and 28 of 58 farmers (48.3%) were producing under different labels with improved animal welfare standards, including IP-Suisse (25/58), Terrasuisse (1/58), Suisse-Garantie (1/58), and Coop Natura Farm (1/58). Farms were located in valley regions (22/58, 38.0%), hill regions (18/58, 31.0%) and mountain regions (18/58, 31.0%).

### Decision-Making Process

**Figure 1:**
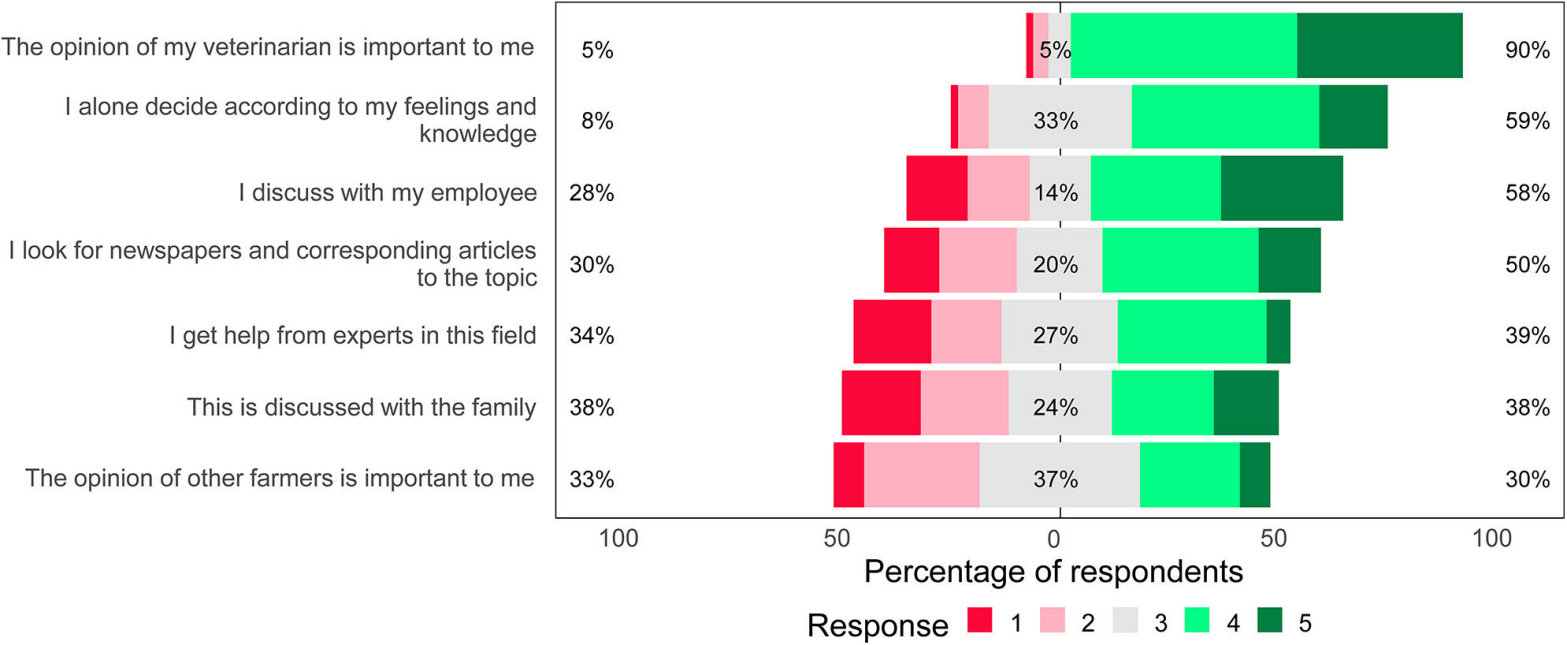
The importance of factors affecting the decision-making of Swiss dairy farmers in dairy cow management. Farmers (*n* = 55–58) ranked their response on a scale from 1 (not a reason) to 5 (a very important reason). The median value of a neutral response is aligned with 0%. The proportions of farmers ranking each factor as negative/less important (1+2), neutral (3), or positive/more important (4+5) are indicated.

Farmers ranked the importance of different factors on their decision-making concerning dairy cow management. The opinion of the veterinarian (median = 4, interquartile range (IQR) = 4–5), and the farmer's own feelings and knowledge (median = 4, IQR = 3–4) were ranked as significantly more important in the decision-making of the farmers regarding dairy cow management than other factors (*P* < 0.001, Kruskal–Wallis) (Figure 1). Work colleagues (median = 3, IQR = 2–4), other experts such as nutritionists (median = 3, IQR = 2–4), and family members (median= 3, IQR = 2–4) were significantly less important in farmer decision-making than other factors.

Farmers were asked about the value of organized meetings and discussion between colleagues and its influence on their decision-making. Thirty-seven of 55 farmers like the idea for organized group meetings with their work colleagues for professional exchange and rated it as important or very important (median = 4, IQR = 3–5). Most (41/55, 74.5%) of the farmers ranked the statement “I can benefit from the meetings” as important or very important (median = 4, IQR = 3.5–5). Furthermore, the majority of respondents (37/55, 67.3%) indicated that they had implemented tips received from other farmers during group meetings (median =4, IQR = 3–4). Time constraint was ranked as less important of a motivating factor for farmers to attend the meetings (median = 2, IQR = 1–3). Moreover, farmers ranked as less important the difficulty of figuring out from different opinions what would be recommendable for them (median = 2, IQR = 1–3).

### Motivation for Reduction of Antimicrobial Use

**Figure 2:**
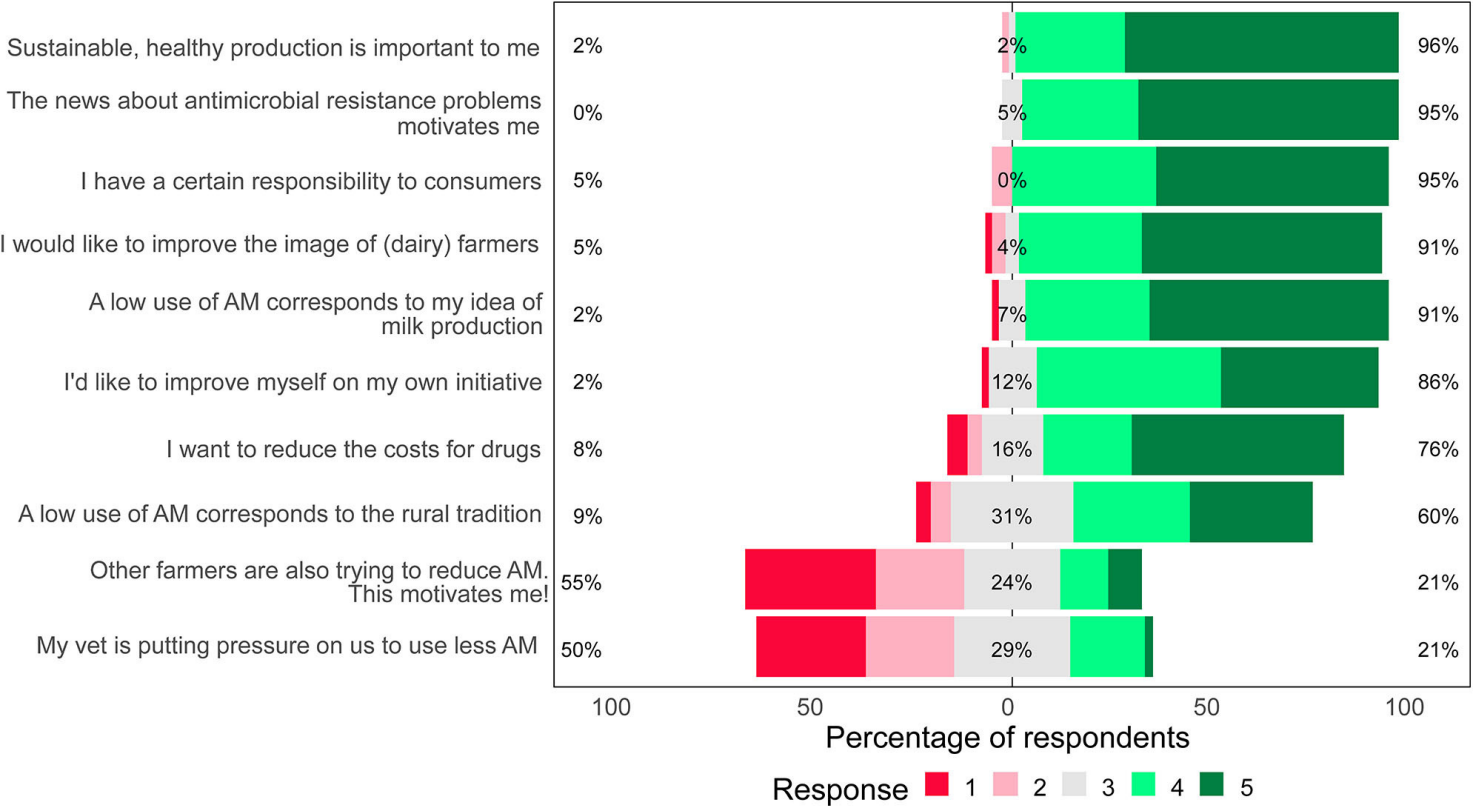
The importance of factors motivating Swiss dairy farmers to reduce antimicrobial use. Farmers (*n* = 58) ranked their response on a scale from 1 (not a reason) to 5 (a very important reason). The median value of a neutral response is aligned with 0%. The proportions of farmers ranking each factor as negative/less important (1+2), neutral (3), or positive/more important (4+5) are indicated (AM, antimicrobials).

Farmers ranked factors that motivate their reduction of antimicrobial use. Respondents ranked two factors as significantly less motivating than the others for reducing antimicrobial use: pressure from the veterinarian (median = 2.5, IQR = 1–3) and awareness that other farmers are trying to reduce antimicrobial use (median = 2, IQR = 1–3) (*P* < 0.001, Kruskal–Wallis) (Figure 2).

### Motivation for Selection of Prevention Strategies

**Figure 3:**
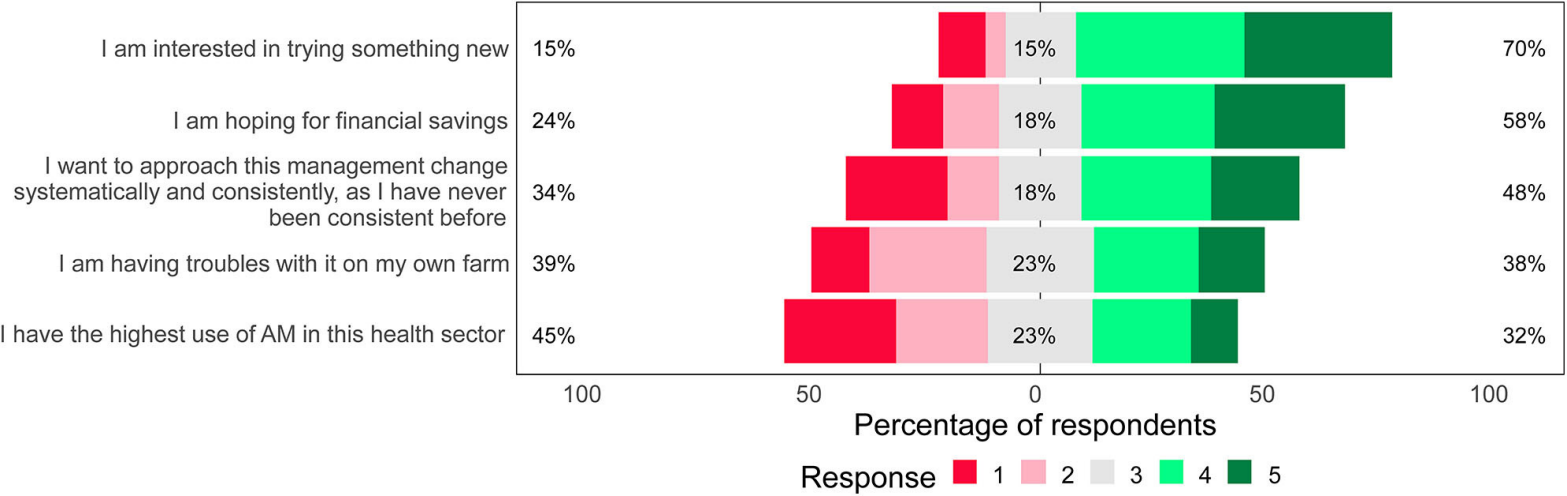
The importance of factors affecting the selection of prevention strategies by Swiss dairy farmers on their farm. Farmers (*n* = 58) ranked their response on a scale from 1 (not a reason) to 5 (a very important reason). The median value of a neutral response is aligned with 0%. The proportions of farmers ranking each factor as negative/less important (1+2), neutral (3), or positive/more important (4+5) are indicated (AM, antimicrobials).

Farmers ranked motivating factors for selecting a specific prevention strategy. Two factors were ranked significantly more positive than other factors: “I want to try something new” (median = 4, IQR = 3–5) and “I want to reduce my costs” (median = 4, IQR = 3–5) (*P* < 0.01, Kruskal–Wallis) (Figure 3). The factor “trying something new” was ranked as a more important reason than reducing costs.

The reported importance of a motivator did not differ significantly based on the health sector for which the farmers wanted to reduce antimicrobial use with the exception of “I have the highest use of antimicrobials in this field” (median = 3, IQR = 2–4), which was a significantly more important reason for reducing antimicrobial use in maintaining udder health compared to uterine health (*p* = 0.04).

### Relationship With the Veterinarian

Farmers indicated they were satisfied with the quality of the consultancy given by their veterinarians (median = 4, IQR = 4–5). They reported that they understood the criticism and suggestions of the veterinarian (median = 4, IQR = 3.75–4) and felt their veterinarians understood their concerns (median = 4, IQR = 3–4). However, only 25.9% (14/54) of the farmers indicated they were willing to pay for good quality, farm-adapted consultancy from veterinarians. Seven of 40 (17.5%) respondents indicated they were not willing to pay extra for this consultancy as they considered it to be already part of paid veterinary services.

## Discussion

In this study we analyzed the responses of a representative cohort of Swiss dairy farmers regarding the factors and people most important in their decision making on the farm, with particular focus on antimicrobial use. Veterinarians were considered a very important factor and therefore appear to have the most influence on farmers with regard to decision-making in dairy cow management. However, veterinarians also were considered as the least motivating factor for farmers for reducing antimicrobial use on their farms. We also found that dairy farmers were highly satisfied with the consultancy provided by veterinarians for facilitating their decision-making. However, only a few farmers indicated they would be willing to pay extra for this service.

The results of our study confirm the high importance of veterinarians and their role on the farm found by other studies (11, 18, 34, 35). Lam et al. showed that the relationship between veterinarian and farmer played an important role in motivating and supporting farmers (22). In addition, we found that the farmers' own feelings and knowledge were an important factor that can influence their own decisions by influencing their intuition. This intuition has a substantial impact on the intention to achieve a certain behavior (18). It would be interesting to investigate further what the intuition of dairy farmers is based on, and how it may positively influence a situation and therefore strengthen the intention to implement a behavior. For example, veterinarians can influence the farmer's experience and knowledge and therefore their attitude and feelings, potentially influencing them to call the veterinarian for consultancy earlier in the future. Such positive influences would help to make the implementation of management changes more successful in practice.

Knowledge acquisition for decision-making via media such as newspapers or scientific journals, was rarely reported as an important reason to make decisions in herd management or antimicrobial use in this study. Due to the relatively high average age of respondents of almost 50 years, the question arises whether the survey might have underestimated the influence of media such as the internet or social media. Younger farmers might consult online search engines to expand their knowledge. In such situations, the danger of misinformation is high, making it crucial for veterinarians to promote high-quality, evidence-based information on antimicrobial use and consultancy.

Farmer responses were mostly neutral or negative with regard to the role of social pressure from family, colleagues or other consultants in decision-making. This suggests that the subjective norm described by the theory of planned behavior has only a minor influence on the intentions of dairy farmers. This finding is in line with the results of a survey of grain farmers and their decision regarding organic or non-organic production (36). In that study farmers were interested in the opinion of individuals within their social environment but did not allow themselves to be pressured by the social environment to make a decision. There is also evidence showing the opposite effect: one of the strongest drivers for livestock farmers to reduce antimicrobial use is the belief that their social and advisory network would approve of them to do so (35).

There are several possible reasons for the low importance attributed by farmers to the role of veterinarians on their motivation to reduce antimicrobial use. First, in Switzerland the change from individual animal medicine to herd-health management and thus toward prevention rather than treatment has been very slow, and veterinarians are often called to a farm only for emergencies (37). Second, in Switzerland only veterinarians are allowed to prescribe and sell antimicrobials, so a significant amount of their revenue is made from drug sales. Under these circumstances, it may be understandable that veterinarians do not push hard to reduce antimicrobial use or increase veterinary consultancy. It also has been reported that veterinarians do not feel motivated by farmers to reduce the use of antimicrobials (37). Independent of the reason for different perceptions, veterinarians, as experts of animal health, should be aware of their responsibility and their potential influence on farmers in making decisions, using antimicrobials, and ultimately helping prevent antimicrobial resistance. Based on our results, there is considerable room for improvement for veterinarians to serve as motivating sources with regard to reducing antimicrobial use on dairy farms.

The ongoing international trend from individual animal medicine toward preventive medicine in food animals is driven by several important factors, including legal regulations, structural change in agriculture, financial pressure, and the demands of consumers and the public (38). The strong upswing in herd-health management in recent years has been due mainly to increasing legal restrictions on the use of antimicrobials; increasing farm sizes and the subsequent decrease in time available to farmers to observe individual animals; and an increasing desire by consumers for improvements in animal welfare and animal health (38, 39). These trends have shifted the veterinary profession more toward consultancy on herd-health management. According to Swiss veterinarians, it is difficult to charge for the increasing consultancy service, although a substantial proportion of working time is allocated to it (37). As shown in the current study, farmers are unknowingly using veterinary services extensively without explicitly being charged for them. For this reason, it is surprising that ~75% of the farmers surveyed were not willing to pay for veterinary consultancy. This may indicate ineffective communication between veterinarians and farmers, where clarifying discussions about paid service are often lacking. Veterinarian communication with farmers that clarifies the benefit of good quality, farm-adapted consultancy, and the value it offers that is worth paying for, could be improved. In Switzerland, the cost of consultancy time often is included in other veterinary services or fees (e.g., as part of milk analysis costs) (37). Lack of transparency in veterinary fees can lead to an unclear situation for farmers who may not be aware that consultancy was provided. Veterinarians have been encouraged to take the initiative to show farmers that there is a need for consultancy to improve their management (22). At the same time, the quality of veterinary consultancy services should stand out from that of others, such as feed consultants, so farmers recognize and are more willing to pay for the added value.

Economic factors were reported to be an important reason for deciding in which health sector farmers want to reduce antimicrobial use. An economically efficient strategy was a stronger reason for farmers to change their management than simply the aim to improve the health situation on their farm, consistent with findings from other studies (12). However, we assume that financial benefits only help to a certain extent to motivate farmer's behavior.

The study population was small, however, the response rate was very high, likely because of the above-average motivation of farmers taking part voluntarily in the ReLait project. We can assume that the participation of farmers with above-average motivation has a major impact on the evaluation of motivation regarding the choice of prevention strategies, but not on the assessment of the role of the veterinarian, the factors influencing the decision-making process, or the motivators that trigger a reduction in antimicrobial use.

By including different farms regardless of herd size, label production, and other farm characteristics, it was assumed that the dairy farmer population was representative of the population in the canton of Fribourg and in Switzerland. The herd size of the farms surveyed was above the Swiss average (22 cows per farm in 2018) (40). It is not clear how this fact may have affected the results of the study. On one hand, it is possible that larger farms have a better relationship with their veterinarian and that veterinarians have a greater influence on farmers, since they have more animals and receive more frequent visits from the veterinarian. On the other hand, the frequency of veterinary visits depends more on the health status of individual animals than on herd size. It can also be argued that larger farms are more professionally managed and therefore more independent from veterinary support for routine procedures such as calf debudding, cow insemination, and obstetrics. Farmers with larger herds may be more economically driven than farmers with small herds who often have a secondary income source. Therefore, farmers with dairy farming as their main occupation are more motivated to reduce antimicrobial use because of costs and because they anticipate possible future restrictions (35). Because the farms participating in the study were larger than the Swiss average, we do not believe that herd size biased the results of this study. However, because respondents were primarily professional farmers, the results may not be generalizable to all other farmers. The proportion of farms producing under organic conditions in this study (8.5%), was comparable to that observed within the canton of Fribourg (6.3%) and of Switzerland as a whole (7.6%) (32, 41). Finally, the landscape zones of farms in this study also were representative of the canton and the country (42). This is important as the prevalence of clinical mastitis—and thus the use of antimicrobials—can vary between farms in different landscape regions in Switzerland (43).

## Conclusions

In this study we showed the importance of the veterinarian's role in Swiss dairy farmers' motivation and decision-making with regard to herd management and antimicrobial use. Farmers and veterinarians alike should be aware of the importance of this relationship and use it to their advantage. Without mutual motivation, it is difficult to work toward a reduction in antimicrobial use. How veterinarians can better motivate farmers to reduce antimicrobial use should be investigated in future studies. We encourage veterinarians to more clearly define the quality and unique contribution of their consultancy from that of other service providers so farmers recognize the added value and are willing to pay for it.

## Data Availability Statement

The dataset used in this study can be found in the Appendix 1 (Supplementary Material). Demographic data (due to privacy issues) and answers of questions not analyzed in this study are not included in the dataset.

## Ethics Statement

According to the Cantonal Ethics Committee for Research, there is no obligation to obtain a license for the questionnaire study presented here [written confirmation with the reference number (BASEC No.) Req-2018-00020]. The reason for this is that the project does not fall under the Human Research Act. For the present study, only farmers participating in this ReLait project (approved by the Federal Office for Agriculture) were interviewed and no ethically reprehensible data were collected.

## Author Contributions

MG, MB, and SD contributed to the conception and design of the study. MG and MB performed the data collection. MG and SD performed the statistical analysis. MG, MB, and SD interpreted the study results. MG wrote the first draft of the manuscript. All authors contributed to manuscript revision, read, and approved the submitted version.

## Conflict of Interest

The authors declare that the research was conducted in the absence of any commercial or financial relationships that could be construed as a potential conflict of interest.

## Supplementary Material

Appendix 1 Shortened data set (without demographic data) as Excel table.
